# Detecting *Leishmania* in dogs: A hierarchical-modeling approach to investigate the performance of parasitological and qPCR-based diagnostic procedures

**DOI:** 10.1371/journal.pntd.0011011

**Published:** 2022-12-16

**Authors:** Tamires Vital, Ana Izabel Passarella Teixeira, Débora Marcolino Silva, Bruna Caroline de Carvalho, Bruno Dallago, Luciana Hagström, Mariana Machado Hecht, Nadjar Nitz, Fernando Abad-Franch

**Affiliations:** 1 Laboratório Interdisciplinar de Biociências, Faculdade de Medicina, Universidade de Brasília, Brasília, Distrito Federal, Brazil; 2 Núcleo de Medicina Tropical, Faculdade de Medicina, Universidade de Brasília, Brasília, Distrito Federal, Brazil; 3 Laboratório de Bem-estar Animal, Faculdade de Agronomia e Medicina Veterinária, Universidade de Brasília, Brasília, Distrito Federal, Brazil; Insitut Pasteur de Tunis, TUNISIA

## Abstract

**Background:**

Domestic dogs are primary reservoir hosts of *Leishmania infantum*, the agent of visceral leishmaniasis. Detecting dog infections is central to epidemiological inference, disease prevention, and veterinary practice. Error-free diagnostic procedures, however, are lacking, and the performance of those available is difficult to measure in the absence of fail-safe “reference standards”. Here, we illustrate how a hierarchical-modeling approach can be used to formally account for false-negative and false-positive results when investigating the process of *Leishmania* detection in dogs.

**Methods/Findings:**

We studied 294 field-sampled dogs of unknown infection status from a *Leishmania*-endemic region. We ran 350 parasitological tests (bone-marrow microscopy and culture) and 1,016 qPCR assays (blood, bone-marrow, and eye-swab samples with amplifiable DNA). Using replicate test results and site-occupancy models, we estimated (a) clinical sensitivity for each diagnostic procedure and (b) clinical specificity for qPCRs; parasitological tests were assumed 100% specific. Initial modeling revealed qPCR specificity < 94%; we tracked the source of this unexpected result to some qPCR plates having subtle signs of possible contamination. Using multi-model inference, we formally accounted for suspected plate contamination and estimated qPCR sensitivity at 49–53% across sample types and dog clinical conditions; qPCR specificity was high (95–96%), but fell to 81–82% for assays run in plates with suspected contamination. The sensitivity of parasitological procedures was low (~12–13%), but increased to ~33% (with substantial uncertainty) for bone-marrow culture in seriously-diseased dogs. *Leishmania*-infection frequency estimates (~49–50% across clinical conditions) were lower than observed (~60%).

**Conclusions:**

We provide statistical estimates of key performance parameters for five diagnostic procedures used to detect *Leishmania* in dogs. Low clinical sensitivies likely reflect the absence of *Leishmania* parasites/DNA in perhaps ~50–70% of samples drawn from infected dogs. Although qPCR performance was similar across sample types, non-invasive eye-swabs were overall less likely to contain amplifiable DNA. Finally, modeling was instrumental to discovering (and formally accounting for) possible qPCR-plate contamination; even with stringent negative/blank-control scoring, ~4–5% of positive qPCRs were most likely false-positives. This work shows, in sum, how hierarchical site-occupancy models can sharpen our understanding of the problem of diagnosing host infections with hard-to-detect pathogens including *Leishmania*.

## Author summary

*Leishmania infantum* is a parasite of dogs and humans that causes a life-threatening disease called visceral leishmaniasis. Detecting dog infections is critical for disease management and prevention, yet error-free diagnostics are unavailable. Here, we illustrate a statistical-modeling approach that can be used to account for the false-negative and false-positive test results that inevitably arise when attempting to detect *Leishmania* in dogs. We used parasitological and molecular testing to study 294 field-sampled dogs from central Brazil. Results from replicate tests and site-occupancy models suggest that molecular testing was ~49–53% sensitive; specificity was high (95–96%) only when very stringent criteria were used to detect and handle possible sample contamination. The sensitivity of parasitological testing was low (~12–13%), but reached ~33% for some samples and dogs. These testing-performance estimates suggest that *Leishmania*-infection frequency was lower than observed–and, in particular, that about 30 seemingly positive dogs were most likely infection-free. Our results show, in sum, how a statistical approach based on hierarchical models can help us better understand the process of detecting elusive microbial pathogens such as *Leishmania*.

## Introduction

The leishmaniases are caused by parasites of the genus *Leishmania*, which are vectored by blood-feeding sandflies [1]. Recent estimates suggest that about 3.8–4.5 million people are infected with *Leishmania* spp. worldwide–and that an average ~750,000 new infections occurred each year between 2010 and 2019 [2]. Visceral leishmaniasis (VL), the most severe form of the disease, is typically fatal if untreated [1]. *Leishmania donovani* causes VL in east Africa and India, whereas *L*. *infantum* causes zoonotic VL (in which transmission involves non-human reservoirs) in parts of the Caucasus and central Asia, across the Mediterranean basin, and in Latin America [1]. Because domestic dogs are the primary reservoir of zoonotic VL [1,3–5], reliably ascertaining whether a dog is infected with *L*. *infantum* is critical both to our understanding of VL epidemiology and to the design, implementation, and evaluation of interventions aiming to prevent human infection [1,3–7]. Because *Leishmania* infection impairs dogs’ health too, accurate diagnosis is also important in veterinary practice [3,4].

There are three main approaches to diagnosing *Leishmania* infection in dogs. Serological tests aim at detecting anti-*Leishmania* antibodies (usually in peripheral-blood or serum samples), parasitological tests aim at detecting *Leishmania* parasites (in, e.g., bone-marrow, lymph-node or spleen aspirates), and PCR-based molecular tests aim at detecting *Leishmania* DNA (in blood or in tissue aspirates as above or using non-invasive samples such as conjunctival eye-swabs) [3–5,8]. Serological tests can have high sensitivities (though never ≡ 100%), yet cross-reactive antibodies against other pathogens can impair specificity [3–5,8–10]. Parasitological tests based on the visualization of *Leishmania* parasites (either directly or after growth in culture media) can have specificities ≅ 100%, yet overall sensitivities may be low [3–5,8]. False-negative results can arise when samples drawn from infected dogs do not contain *Leishmania* parasites or when the test fails to detect parasites that are present in the sample [11,12]. Finally, DNA-based methods can have high sensitivites [4,5,13–16]; in practice, however, the parasites’ DNA may not be present in all biological samples drawn from infected hosts, and any PCR can fail to amplify the target DNA stretch (e.g., because of polymerase inhibition or variation in primer-binding sequences) even when it is present in the sample [11,17]. Suitably designed primers help ensure that DNA-based tests have high specificities, but stringent protocols are required to avoid (or detect) sample contamination with *Leishmania* DNA amplicons [18].

This overview shows that, as with any other pathogen and host [11,12,19,20], the problem of detecting *Leishmania* in dogs can be a complex undertaking [3–5,8–10]. It may become even more complicated if extrinsic factors can affect test performance [3–5,8–10,21–24]. For example, higher *Leishmania* loads in seriously-diseased dogs can result in overall higher sensitivity of parasitological and DNA-based diagnostic procedures [21,22]. In addition, and importantly, test-performance estimates derived from laboratory studies done under highly-controlled conditions (with, e.g., samples spiked with parasites or their DNA) often differ substantially from those derived from field studies [25–28]. A key reason for this difference is that, in laboratory studies, test-performance metrics are usually computed based on samples of known infection status, whereas in field studies the samples may, or may not, contain the target for detection [11,12]. More generally, a crucial (yet seldom discussed) point is that test sensitivity and specificity are computed using different denominators, and under different underlying assumptions, across various common types of diagnostic test-performance studies (Table 1). Thus, the meaning of words like “sensitivity” and “specificity” effectively changes across (and at times even within) studies, and this makes direct comparisons of quantitative results potentially misleading.

**Table 1:** Testing tests: In many common types of diagnostic test-performance studies, the two key performance metrics (sensitivity and specificity) are computed using different denominators and under different, seldom explicit assumptions.

Type of study^a^	Metric	Numerator (observations)	Denominator (“truth”)	Major assumptions^b^
Spiked samples	Sensitivity	Samples positive by index test^c^	Samples spiked with the target for detection	Spiking effective
	Specificity	Samples negative by index test	Samples not spiked with the target for detection	No cross-spiking
Experimental infection 1	Sensitivity	Samples positive by index test	Samples drawn from experimentally infected hosts	Infection procedure effective; perfect target availability^d^
	Specificity	Samples negative by index test	Samples drawn from control hosts (not experimentally infected)	No cryptic infection
Experimental infection 2	Sensitivity	Samples positive by index test	Samples drawn from experimentally infected hosts and positive by reference standard^e^	Infection procedure effective; perfect reference standard
	Specificity	Samples negative by index test	Samples drawn from control hosts (not experimentally infected) and negative by reference standard	No cryptic infection; perfect reference standard
Natural infection 1	Sensitivity	Samples positive by index test	Samples positive by reference standard	Perfect reference standard
	Specificity	Samples negative by index test	Samples negative by reference standard	Perfect reference standard
Natural infection 2	Sensitivity	Samples positive by index test	Samples drawn from reference standard-positive hosts (but samples not tested by reference standard)	Perfect reference standard and perfect target availability
	Specificity	Samples negative by index test	Samples drawn from reference standard-negative hosts (but samples not tested by reference standard)	Perfect reference standard and perfect target availability
Natural infection 3	Sensitivity	Samples positive by index test	Samples drawn from reference standard-positive hosts and, in addition, positive by reference standard (a “double-testing tactic”–first hosts, then samples)	Perfect target availability at host level; perfect reference standard at both host and sample levels
	Specificity	Samples negative by index test	Samples drawn from reference standard-negative hosts and, in addition, negative by reference standard (a “double-testing tactic”–first hosts, then samples)	Perfect target availability at host level; perfect reference standard at both host and sample levels

^a^ Studies based on panels of samples typically use one of the tactics already summarized in this Table, depending on the origin of the samples in the panels

^b^ Beyond target integrity (e.g., DNA or antibodies not degraded) and absence of sample contamination

^c^ The “index test” is the diagnostic test under evaluation–i.e., the test whose sensitivity and specificity investigators aim to estimate

^d^ “Target availability” refers to the presence of the target for detection by the index test (e.g., pathogens, DNA, antigens, antibodies, or other biomarkers) in the actual sample used for testing

^e^ The “reference standard” is a procedure used to classify hosts or samples as “positive” or “negative”; it may comprise one or several diagnostic tests, expert clinical opinion, or a combination thereof–and the results, when available, are effectively interpreted as revealing the true state (“positive” or “negative”) of the host or sample

Most uncertainties about the performance of different *Leishmania* diagnostic procedures ultimately stem from the fact that, in practice, there is no fail-safe “reference-standard” method with 100% sensitivity and 100% specificity [3–5,8–10,13–16,21–26,29–31]. Veterinary doctors, for example, have to rely on imperfect diagnostic tools to make clinical decisions–e.g., whether to treat, when to stop treatment, or, in some settings, whether to euthanize positive dogs (e.g., [8,21,22]; see also [32]). In epizootiological studies, a common approach to dealing with diagnostic uncertainty is to simply ignore it. Often, the results of a single test are used to classify subjects as infected or not, even if this entails assuming that (a) all samples drawn from infected subjects contain the target for detection and (b) the test has 100% sensitivity and 100% specificity [11]. When two or more tests are used and subjects with at least one positive test result are classified as infected, assumption (b) is implicitly changed to state that all tests have 100% specificity and the sensitivity of the several tests combined is also 100%. Although the assumptions we have just outlined are almost never justified, this approach is surprisingly widespread (e.g., [6,14,33–35]).

Similar complications arise with the use of “reference standards” for investigating the performance of “index” tests. Single-test reference standards again imply assuming perfect performance of the reference test; because this is patently implausible, “composite reference standards” (in which the results of two or more tests are used to classify individuals as infected or not; see Table 1) are considered preferable [10,36]. In reality, though, composite standards combine the imperfect (and often unknown) sensitivites and specificities of the various tests used to compose them. For example, a composite standard including one parasitological test (100% specific and 45% sensitive) and two molecular tests (both 95% specific and 70% sensitive) should be expected to misclassify as infected about 10% of uninfected hosts and as uninfected about 5% of infected hosts. These reservations apply to any test evaluation in which samples are classified as positive or negative based on a (less-than-perfect) reference standard and then submitted to the (also imperfect) index test(s) (cf. [10,37,38]; see also Table 1).

In this work, we illustrate a different approach to investigating the performance of parasitological and molecular methods widely used to diagnose *Leishmania* infection in dogs. The approach is based on a hierarchical-modeling framework developed to study wildlife site-occupancy when detection failures (i.e., false negatives) and species misidentification (i.e., false positives) can both occur [39,40]. In principle, these models can find application in any pathogen-detection problem involving imperfect diagnostic methods [19,20,41,42]; for example, we have previously used this approach to tackle the problem of detecting *Trypanosoma cruzi*, the agent of Chagas disease, in its insect vectors [43]. In the present study, we focus on the process of diagnosing *Leishmania* infections in field-sampled dogs of unknown infection status. Specifically, we set to (i) estimate the sensitivity and specificity of two parasitological and and three DNA-based diagnostic procedures; (ii) further explore the potential value of non-invasive biological samples for DNA-based diagnosis; (iii) determine whether and how dog clinical condition affects the sensitivity of the different diagnostic procedures; and (iv) derive statistical estimates of *Leishmania* infection frequency that formally account for the uncertainties inherent to the diagnostic process. More generally, we illustrate how hierarchical models can improve our understanding of the naturally hierarchical problem of diagnosing infection by hard-to-detect pathogens including *Leishmania*.

## Methods

### Ethics statement

All the procedures described below were reviewed and approved by the Ethics Committee on Animal Use of the Instituto de Ciências Biológicas, Universidade de Brasília, Brazil (CEUA UnBDoc number 11253/2015).

### Dogs and samples

**Fig 1:**
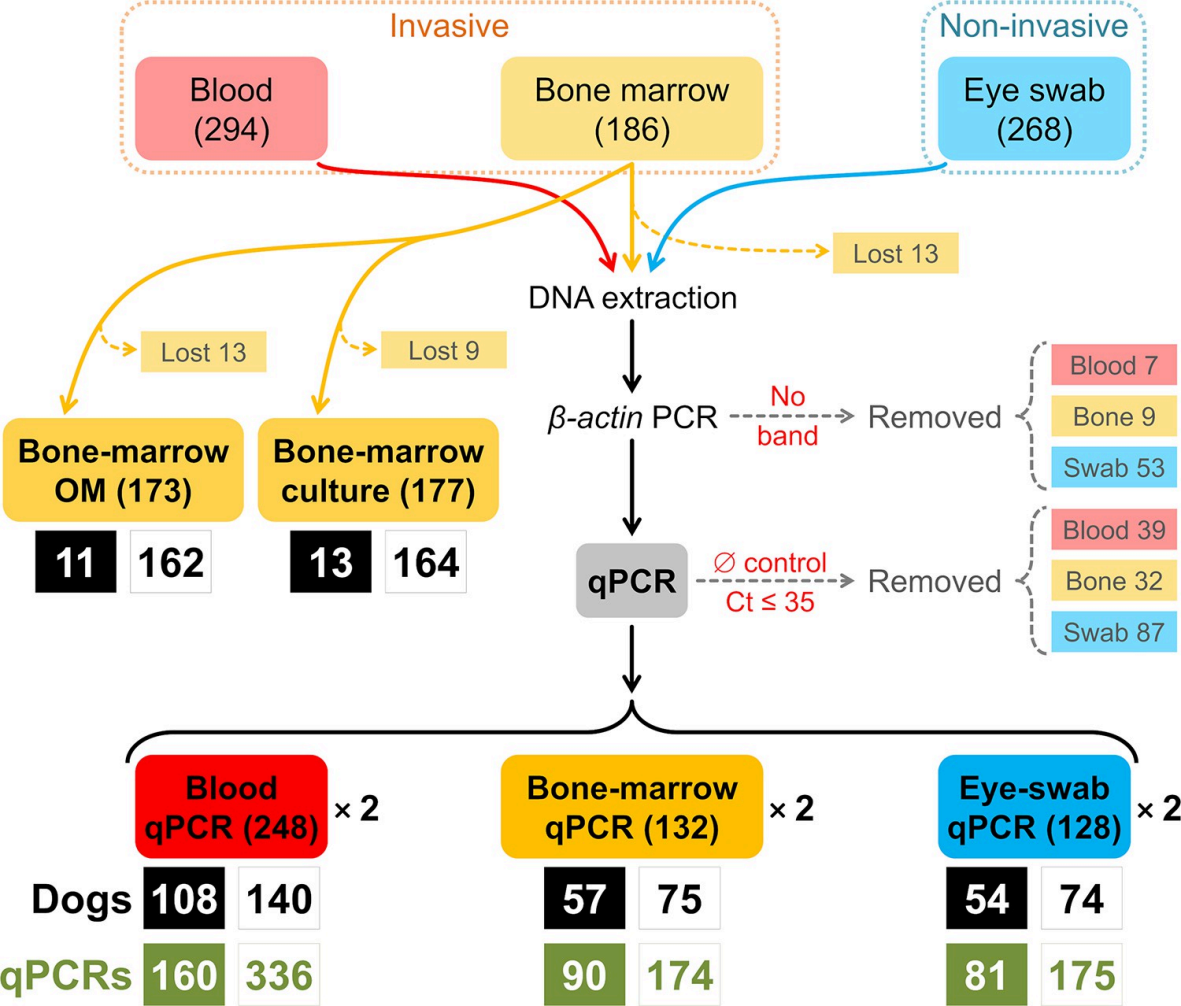
Detecting *Leishmania* in dogs: Schematic of the sampling-testing process. Invasive samples were taken from 294 (peripheral blood) and 186 dogs (bone-marrow aspirates), and non-invasive samples (conjunctival eye swabs) from 268 dogs. Some bone-marrow samples were lost before processing (indicated by “Lost”); 173 were tested by optical microscopy (“OM”), 177 by culture, and 173 were submitted to DNA extraction for quantitative PCR (“qPCR”). Blood and eye-swab samples were all submitted to DNA extraction. We PCR-amplified the constitutive dog *β-actin* gene and removed samples yielding no amplification (indicated by “No band” and then “Removed” in the “*β-actin* PCR” step). The rest of samples had amplifiable DNA and were used for duplicate *Leishmania* kDNA qPCR assays; of these, some were not considered further because there was DNA amplification with cycle-to-threshold (Ct) values ≤ 35 cycles in the negative and/or blank controls (“∅”) of their qPCR plates (indicated by “∅ Ct ≤ 35” and then “Removed” in the “qPCR” step). The results of qPCRs run on plates in which all negative and blank controls had either no amplification or high-Ct amplification (> 35 cycles) were used for our analyses. These correspond to duplicate qPCRs run on blood (248 dogs, 496 qPCRs), bone-marrow (132 dogs, 264 qPCRs) and eye-swab samples (128 dogs, 256 qPCRs). The results of each diagnostic procedure are shown as the number of positive dogs (black boxes) and qPCRs (green boxes) plus the number of negative dogs and qPCRs (white boxes).

This study was done in the Federal District of Brazil, where canine VL is endemic [36,44]. We sampled 294 resident domestic dogs between October 2015 and May 2017. Our systematic-sampling scheme aimed at studying the dogs living in one house per city block, provided that (i) the owner was at least 18 years old and read and signed an informed consent form and (ii) the dog was older that four months and safe to handle for specimen sampling (see details in [44]); a few blocks were skipped either because no house had dogs or because dog owners were absent or refused to participate (cf. [44]). At the time of sampling, a veterinarian used the clinical-scoring system described in [36] to classify each dog as “apparently healthy” (clinical score 0), “mildly diseased” (clinical score 1 to 4), or “seriously diseased” (clinical score ≥ 5) (see [36] and also [23]). We drew three types of samples: peripheral blood, bone-marrow aspirates, and conjunctival eye-swabs (see below). Fig 1 summarizes the sampling and testing process.

### Diagnostic procedures

#### Sampling

Peripheral-blood samples (~3 mL) were drawn from the cephalic, saphenous, or jugular vein, and bone-marrow aspirates (~1 mL) from the sternum. These invasive samples were stored in Vacutainer K2EDTA sterile tubes (BD, Franklin Lakes, NJ); tubes were kept in dry ice during transportation to the laboratory and stored in −20°C freezers until DNA extraction. Non-invasive conjunctival eye-swabs were taken with sterile cotton swabs (Labor Import, Osasco, Brazil); the swab tips were cut and stored in sterile 1.5 mL microtubes (Kasvi, São José dos Pinhais, Brazil) without adding any solution. The microtubes were kept/stored as above.

#### Processing and scoring: Parasitological tests

*Optical microscopy (OM)*. We prepared two optical-microscopy slides with ~20 μL of each dog’s bone-marrow aspirate immediately after collection. The slides were fixed with methanol and conditioned for transportation; in the laboratory, they were stained with Giemsa (Merck, Dramstadt, Germany) or Panotico Instant Prov (Newprov, Pinhais, Brazil) and observed under a Leica DM500 light microscope with immersion oil and 100× magnification [45]. We read 1000 fields per slide and scored the OM test as positive if at least one *Leishmania* amastigote was unambiguously identified in at least one slide.

*Bone-marrow culture (BMC)*. Using sterile insulin syringes, we seeded ~20 μL of bone-marrow aspirate into each of two BD Vacutainer tubes containing 5 mL of a base agar preparation (no. 2, Difco 0696–17; BD, NJ), 10% defibrinated rabbit blood (added after agar fusion), and gentamicin 100 μg/mL (Schering-Plough, Kenilworth, NJ) [46]. The tubes had been sealed and stored in an oven at room temperature for 24 h to check for bacterial or fungal contamination, and then stored at 4°C until use. Seeded culture tubes were checked for *Leishmania* every two days (over a 30-day period) using a Leica DMi1 inverted optical microscope [46]. This BMC diagnostic procedure was scored as positive when at least one *Leishmania* promastigote was unambiguously identified in at least one inverted-microscope reading.

#### Processing and scoring: qPCR

DNA was isolated from blood and bone-marrow samples with the Wizard Genomic DNA Purification kit (Promega, Madison, WI); we followed the manufacturer’s protocol for whole blood. Eye-swab DNA was isolated from swab tips with the Biopur Mini Spin Plus kit (Biometrix, Atuba, Brazil); we slightly modified the manufacturer’s protocol (see S1 Protocol). We determined DNA concentrations using a NanoVue Plus spectrophotometer (GE Healthcare, Chicago, IL). For qPCR we used *Leishmania* kDNA minicircle-specific primers [47]; each reaction included 50 ng of template DNA, 0.2 μM of each primer, and 10 μL of 1× Power SYBR Green PCR Master Mix (Applied Biosystems, Foster City, CA) in a final volume of 20 μL. After 12 min at 94°C, reactions ran for 40 cycles of 94°C for 30 sec, 55°C for 30 sec, and 72°C for 30 sec. Melting curves were calculated at 95°C for 5 sec, 50°C for 15 sec, and 95°C for 5 sec. All qPCRs were run in duplicate in MicroAmp optical 96-well reaction plates (Thermo Fischer Scientific, Waltham, MA) using Applied Biosystems’ QuantStudio 3. A standard curve was obtained using DNA of *Leishmania infantum* (strain MCER/BR/79/M6445) serially diluted from 5×10^1^ to 5×10^−3^ ng/μL.

*Quality control*. We first verified that DNA extractions had yielded good-quality DNA by PCR-amplifying, in each sample, the dogs’ constitutive *β-actin* gene (cf. [48]). All *Leishmania* qPCR runs included (i) two negative controls, one with the reaction mix but without DNA (blank) and the other with DNA extracted from a culture of uninfected human embryonic kidney (HEK) cells, and (ii) two positive controls with *L*. *infantum* DNA at, respectively 5×10^1^ and 5×10^−3^ ng/μL. To establish melting temperatures (Tm), we amplified DNA from *L*. *infantum* (MCER/BR/79/M6445; 82.5±0.5°C), *L*. *amazonensis* (IFLA/BR/67/PH8; 82.4±0.5°C), *L*. *braziliensis* (MHOM/BR/75/M2483; 80.4±0.5°C), and *L*. *guyanensis* (M44142; 80.4±0.5°C).

*Scoring*. Following standard practice, we scored as positive for *Leishmania* sp. any qPCR assay that (i) yielded amplification of the kDNA target with a cycle-to-threshold (Ct) value ≤ 35.0 cycles and a Tm in the 80.0–83.0°C range typical of *Leishmania* (see above), and (ii) was run on a qPCR plate in which none of the negative or blank control assays yielded DNA amplification with Ct ≤ 35.0 cycles.

#### The data

Our data reflect the results of (i) duplicate qPCRs run on invasive (blood and bone-marrow) and non-invasive (eye-swab) samples, and (ii) two parasitological tests–direct optical microscopy of bone-marrow aspirates (OM) and inverted optical microscopy of bone-marrow cultures (BMC) (Fig 1 and S1 Dataset). The “targets for detection” are *Leishmania* parasites in the case of OM and BMC and *Leishmania* DNA in the case of qPCR. Parasitological-test results were our “reference standard” for specificity–but, importantly, not for sensitivity. This means that, whenever a *Leishmania* parasite was morphologically identified by OM or BMC, that dog was regarded as infected, and the result of that individual test was considered unambiguous (and coded “2”). The results of other tests, whether negative or positive, were all regarded as ambiguous; we now briefly summarize the rationale and coding scheme for each observed outcome.

1. A *negative test result* (coded “0”) may arise from one of two processes: either (i) the dog was not infected or (ii) the dog was infected, yet the test yielded a (possibly wrong) negative result. More specifically, if a dog is infected, a negative test result can arise from (a) failure of the test to detect a target (parasites or their DNA) that is present in the sample (a “true” false-negative test); or (b) absence of the target from the sample submitted to the test–a “false” false-negative, in the sense that the test gave the correct answer (“negative”) at the sample level (“target unavailable”), which is what a specific test is expected to do [11,12]. The problem, therefore, was not with the test, but rather with the overall diagnostic procedure–which failed to provide the correct answer (“positive”) at the dog level (“infected”). Our aim in this work was to estimate the “clinical sensitivity” of the whole diagnostic procedure involving each test–from the drawing of the sample to the scoring of test results. It is easy to see that such estimates would only equal true test sensitivity if sample-level target availability were 100%. Because we can safely assume that availability of *Leishmania* parasites (or their DNA) in any given sample drawn from an infected dog is, on average, <100%, true test sensitivities are most likely higher, on average, than our whole-diagnostic-procedure estimates [11,12]. Importantly, however, our “whole-procedure estimates” directly reflect what practitioners actually see when they investigate *Leishmania* infection (in dogs or any other host species) under field conditions (i.e., clinical sensitivity); they are also what most field studies report, typically as “test sensitivity”–under the strong, yet almost invariably unstated assumption of 100% sample-level target availability [11,12] (see Table 1).
2. A *positive qPCR result* (coded “1”) may also arise from two processes: (i) the dog was infected or (ii) it was not, yet the test yielded a (wrong) positive result. Such a wrong result may, in turn, arise from (a) amplification of DNA from some non-target organism present in the sample or (b) contamination of the sample with “foreign” *Leishmania* DNA. Note that this latter event can hardly be seen as a “true” false-positive at the sample level; after all, the test detected *Leishmania* DNA (test “positive”) in a sample that indeed contained it (“target available”), which is what a sensitive test is expected to do [11]. It is, however, a false-positive result at the (“uninfected”) dog level–and the problem lies, again, in the diagnostic procedure as a whole, including the various steps of sample processing [11]. We therefore re-emphasize that our present assessment involves, as our title indicates, the performance of qPCR-based “diagnostic procedures”, and not of the qPCRs themselves. We also stress that we did not expect amplification of DNA from non-target (non-*Leishmania*) organisms to be of any practical importance–the primers and protocols we used should guarantee that the odds of such an event are effectively negligible [47]. Accordingly, we considered that false-positive results, if any, would in all likelihood be due to sample contamination–and would therefore be, in that sense, “false” false-positives.

We note that not all dogs underwent the full set of eight possible diagnostic procedures (three duplicate qPCRs plus OM and BMC) (see Fig 1). The hierarchical models upon which we base inference (see below) accommodate such missing results (coded “-”) by simply skipping them in the computation of the likelihood–with the natural side-effect of increasing uncertainty about model-based estimates [49].

In what follows, we will refer to “diagnostic procedures” in general as “DPs”; for simplicity, though, we will use “qPCR” for qPCR-based procedures, “OM” for OM-based procedures, and “BMC” for BMC-based procedures.

### Data analyses

We first described our data in tables and graphs, and conducted exploratory analyses to get an initial sense of whether and how DP results (test-level and dog-level) varied across sample/tissue types and among dogs in different clinical condition. We fitted bivariate generalized linear models (GLMs; binomial family, logit link function) with the *glmmTMB* 1.0.1 package [50] in R 3.6.3 [51]; the dependent variable indexed whether each dog had been positive in at least one DP (coded “1”) or not (coded “0”). To summarize the patterns of DP co-positivity and co-negativity, we built a five-set Venn diagram with the *VennDiagram* 1.6.10 R package [52].

In a second, inferential step, we used “multiple detection-state” site-occupancy models [40] to get statistical estimates of DP sensitivity and specificity. These hierarchical models formally account for the facts that any DP can yield a false-negative result and qPCR assays may, in addition, yield some false-positive results [53,54]. As noted above, we assume 100% specificity for parasitological DPs based on the direct visualization of *Leishmania* parasites by expert staff. Finally, the models yield corrected estimates of *Leishmania* infection frequency in our dog sample. We fitted our models in Presence 2.13.11 [55], and compared model performance using second-order Akaike’s information criterion scores (AICc) and Akaike weights (AICw) [56]. For any given model set, models with smaller AICc scores perform better; since AICw values sum to 1.0, each model’s weight can be interpreted as the “weight of evidence” in favor of that model [56]. AICw values were also used to weight model-specific parameter values during information-theoretic model averaging [56]. Further details on our modeling strategy can be found in [43].

We analyzed our data in four consecutive “modeling rounds”. The first round corresponds to our original plan for data analysis; the results of this round, however, suggested that a reappraisal was necessary (see below). The first-round model set comprised 11 models, each representing a specific, *a priori* hypothesis about the performance of each DP. For qPCRs, our hypotheses are about clinical sensitivity and specificity; for parasitological test-based DPs (OM and BMC) our hypotheses refer only to sensitivity (Table 2). Model details are provided in S1 Table (model structure; AICc scores and associated metrics; number of parameters; and deviance) and S2 Table (top-model and model-averaged estimates, with SEs).

**Table 2:** Performance of parasitological and molecular diagnostic procedures (DPs) for detecting *Leishmania* infection in dogs: Models and associated hypotheses.

Modeling round/model	Hypothesis^a^
First round	
{1} Null	All DPs have the same sensitivity and all qPCRs have the same specificity, irrespective of tissue sample (blood, bone-marrow, or eye-swab)
{2} Basic (top 1^st^ round)	Sensitivity differs between qPCRs and parasitological DPs (with the expectation that the former will turn out to be more sensitive than the latter); all qPCRs have the same sensitivity and specificity
{3} Basic-plus	Each DP (qPCR, OM, and BMC) has its own, distinct sensitivity; all qPCRs have the same sensitivity and specificity
{4} Tissue	qPCR sensitivity depends on tissue sample, but specificity is common; parasitological DPs have the same sensitivity
{5} Tissue-plus	qPCR sensitivity depends on tissue sample, but specificity is common; OM and BMC have different sensitivities
{6} Invasive-sampling	qPCR sensitivity differs between invasive (blood or bone-marrow) and non-invasive (eye-swab) samples, but specificity is common to all qPCRs; parasitological DPs have the same sensitivity
{7} Invasive-sampling-plus	qPCR sensitivity differs between invasive and non-invasive samples, but specificity is common to all qPCRs; OM and BMC have different sensitivities
{8} Invasive-sampling-specificity	qPCR sensitivity and specificity both differ between invasive and non-invasive samples; parasitological DPs have the same sensitivity
{9} Invasive-sampling-specificity-plus	qPCR sensitivity and specificity both differ between invasive and non-invasive samples; OM and BMC have different sensitivities
{10} Full	qPCR sensitivity and specificity both depend on tissue sample; parasitological DPs have the same sensitivity
{11} Full-plus	qPCR sensitivity and specificity both depend on tissue sample; OM and BMC have different sensitivities
Second round (with SC)	
{12–22} (First-round models-SC)	As {1–11} above, yet adding “qPCR specificity decreases by the same average amount in samples run on plates with suspected contamination”
{13} Basic-SC (top 2^nd^ round)	Sensitivity differs between qPCRs and parasitological DPs (with the expectation that the former will turn out to be more sensitive than the latter); all qPCRs have the same sensitivity and specificity; qPCR specificity decreases by the same average amount in samples run on plates with suspected contamination
{23} Invasive-sampling-specificity-SC	qPCR sensitivity and specificity both differ between invasive (blood or bone-marrow) and non-invasive (eye-swab) samples; specificity decreases by different amounts in invasive and non-invasive samples run on plates with suspected contamination; parasitological DPs have the same sensitivity
{24} Invasive-sampling-specificity-SC-plus	qPCR sensitivity and specificity both differ between invasive and non-invasive samples; specificity decreases by different amounts in invasive and non-invasive samples run on plates with suspected contamination; OM and BMC have different sensitivities
{25} Full-SC	qPCR sensitivity and specificity both depend on tissue sample (blood, bone marrow, eye swab); specificity decreases by different amounts in different tissue samples run on plates with suspected contamination; parasitological DPs have the same sensitivity
{26} Full-SC-plus	qPCR sensitivity and specificity both depend on tissue sample; specificity decreases by different amounts in different tissue samples run on plates with suspected contamination; OM and BMC have different sensitivities

DP, diagnostic procedure; qPCR, diagnostic procedure based on quantitative real-time PCR; OM, diagnostic procedure based on direct optical microscopy of bone-marrow samples; BMC, diagnostic procedure based on bone-marrow aspirate culture; SC, suspected contamination of qPCR plates

^a^ Note (i) that sensitivity and specificity refer to the whole diagnostic procedure (DP), from the drawing of the sample to the scoring of the test (i.e., “clinical sensitivity” and “clinical specificity”); and (ii) that in all models specificity is assumed perfect (100%) for parasitological DPs involving direct visualization of *Leishmania* parasites

Analysis of the first-round model set revealed lower-than-expected qPCR specificity estimates (cf. [43]), with model-averaged values consistently below 94% (see **Results**). This led us to suspect that some qPCR wells might have become contaminated with *Leishmania* DNA [53]. Since, unfortunately, the samples were not available for retesting, we set to model the possible effects of contamination on qPCR specificity [11]. For this, we rechecked all qPCR results, including the amplification and melting curves of all samples and all positive, negative, and blank controls. We then noted whether or not each individual qPCR result came from a plate in which any of the negative or blank controls had any signs of possible contamination. Specifically, for any given plate we suspected contamination when any negative/blank control had (i) some signal of DNA amplification, even with Ct > 35 cycles, and (ii) a melting curve at least roughly compatible with that expected for *Leishmania* DNA.

To investigate the potential effects of suspected contamination on qPCR performance estimates, we then fitted a “second-round” set of models in which qPCR specificity was allowed to vary as a function of a “suspected contamination” covariate (“SC”) indicating whether each qPCR result came from a plate with (SC = 1) or without (SC = 0) signs of possible contamination (as defined above; see S1 Table and S1 Dataset). We refitted the 11 first-round models after adding the SC covariate, and tested four further specifications in which the effects of suspected plate contamination could be either (i) common to qPCRs run on different samples (invasive *vs*. non-invasive) or tissues (blood, bone marrow, eye swab), or (ii) sample/tissue-specific (Table 2). We compared the performance of the 26 models in the second-round set using AICc scores; we expected (a) that models with the SC covariate would outperform those without it, and (b) that SC effects should be common to all samples/tissues. The results of the second modeling round confirmed these two expectations (S1 Table), and showed that qPCR specificity fell from a realistic 94–96% [43] in samples from plates without signs of contamination to a poor ~85% in samples run on plates with suspected contamination (see **Results**).

With this information, we finally returned to our original aim of determining whether DP sensitivity would vary among dogs in different clinical condition; we had no reason to believe that dog condition would affect qPCR specificity. Using the top-performing (lowest-AICc) model from the second-round set (model {13} in Table 2), we investigated the possible influence of dog clinical condition on test sensitivity by fitting six further models in which DP sensitivity varied depending on whether a dog was (1) apparently healthy (*vs*. mildly or seriously diseased) or (2) seriously diseased (*vs*. healthy-looking or mildly diseased). We tested these two covariates for (i) common effects across DPs, (ii) different effects for qPCRs and parasitological DPs, and (iii) different effects for each DP (qPCR, OM, BMC). Again, we used AICc to compare the performance of all 32 models in this “third-round” set (see S1 Table). We found that the top-performing model (model {32} in Table 3) distinguished the effects of the “seriously diseased” dog condition on each DP, but the slope coefficient estimates for qPCR and OM were effectively indistinguishable from zero, whereas the BMC coefficient was positive (see **Results**). We therefore included, in a “final round” of modeling, a simplified version of model {32} in which the “seriously diseased” dog condition affected BMC sensitivity only; for completeness, we also tested the effects of this condition on the sensitivities of parasitological DPs only, OM only, and qPCR only (Table 3). Finally, we fitted four additional, more complex versions of the top-ranking model in this final-round model set to test for (i) possible differences in DP sensitivity across all three clinical-condition classes and (ii) possible clinical-condition effects on the odds of dog infection. The full model set, hence, comprised 40 models (S1 Table).

**Table 3:** Influence of dog clinical status on the performance of diagnostic procedures (DPs) for detecting *Leishmania*: Models and associated hypotheses.

Modeling round/model	Hypothesis^a^
Third round	
{27} Basic-SC-healthy common	Sensitivity differs between qPCRs and parasitological DPs; all qPCRs have the same sensitivity and specificity; qPCR specificity decreases by the same average amount in samples run on plates with suspected contamination; and DP sensitivity changes (likely decreases) by the same average amount in healthy-looking relative to other dogs
{28} Basic-SC-healthy qPCR/parasitological	Sensitivity differs between qPCRs and parasitological DPs; all qPCRs have the same sensitivity and specificity; qPCR specificity decreases by the same average amount in samples run on plates with suspected contamination; qPCR and parasitological DP sensitivities change by different amounts in healthy-looking relative to other dogs
{29} Basic-SC-healthy qPCR/OM/BMC	Sensitivity differs between qPCRs and parasitological DPs; all qPCRs have the same sensitivity and specificity; qPCR specificity decreases by the same average amount in samples run on plates with suspected contamination; qPCR, OM, and BMC sensitivities change by different amounts in healthy-looking relative to other dogs
{30} Basic-SC-serious disease common	Sensitivity differs between qPCRs and parasitological DPs; all qPCRs have the same sensitivity and specificity; qPCR specificity decreases by the same average amount in samples run on plates with suspected contamination; and DP sensitivity increases by the same average amount in seriously diseased relative to other dogs
{31} Basic-SC-serious disease qPCR/parasitological	Sensitivity differs between qPCRs and parasitological DPs; all qPCRs have the same sensitivity and specificity; qPCR specificity decreases by the same average amount in samples run on plates with suspected contamination; qPCR and parasitological DP sensitivities increase by different amounts in seriously diseased relative to other dogs
{32} Basic-SC-serious disease qPCR/OM/BMC (top 3^rd^ round)	Sensitivity differs between qPCRs and parasitological DPs; all qPCRs have the same sensitivity and specificity; qPCR specificity decreases by the same average amount in samples run on plates with suspected contamination; qPCR, OM, and BMC sensitivities increase by different amounts in seriously diseased relative to other dogs
Final round	
{33} Basic-SC-serious disease qPCR	Sensitivity differs between qPCRs and parasitological DPs; all qPCRs have the same sensitivity and specificity; qPCR specificity decreases by the same average amount in samples run on plates with suspected contamination; qPCR (but not parasitological DP) sensitivity increases in seriously diseased dogs
{34} Basic-SC-serious disease parasitological DPs	Sensitivity differs between qPCRs and parasitological DPs; all qPCRs have the same sensitivity and specificity; qPCR specificity decreases by the same average amount in samples run on plates with suspected contamination; parasitological DP (but not qPCR) sensitivity increases in seriously diseased dogs
{35} Basic-SC-serious disease OM	Sensitivity differs between qPCRs and parasitological DPs; all qPCRs have the same sensitivity and specificity; qPCR specificity decreases by the same average amount in samples run on plates with suspected contamination; OM (but not other DP) sensitivity increases in seriously diseased dogs
{36} Basic-SC-serious disease BMC (top final round)	Sensitivity differs between qPCRs and parasitological DPs; all qPCRs have the same sensitivity and specificity; qPCR specificity decreases by the same average amount in samples run on plates with suspected contamination; BMC (but not other DP) sensitivity increases in seriously diseased dogs
{36b} Basic-SC-all dog condition classes BMC	Same as {36} but with a three-level disease-condition covariate (healthy, mildly diseased, seriously diseased) on BMC
{36c} Basic-SC-serious disease BMC and infection	Same as {36} but with the seriously diseased condition affecting infection probability
{36d} Basic-SC-serious disease BMC-healthy infection	Same as {36} but with the healthy-looking condition affecting infection probability
{36e} Basic-SC-serious disease BMC-all dog condition classes infection	Same as {36} but with a three-level disease-condition covariate (healthy, mildly diseased, seriously diseased) on infection probability

DP, diagnostic procedure; qPCR, diagnostic procedure based on quantitative real-time PCR; OM, diagnostic procedure based on direct optical microscopy of bone-marrow samples; BMC, diagnostic procedure based on bone-marrow culture; SC, suspected qPCR plate contamination

^a^ Note (i) that sensitivity and specificity refer to the whole diagnostic procedure (DP), from the drawing of the sample to the scoring of the test (i.e., “clinical sensitivity” and “clinical specificity”); and (ii) that in all models specificity is assumed perfect (100%) for parasitological DPs involving direct visualization of *Leishmania* parasites

We base inference on three interrelated outcomes of the analytical strategy (all “rounds” of modeling) described above: (i) the performance of different models, as gauged by AICc and AICw–which together provide a measure of the relative support that the hypothesis associated with each model (Tables 2 and 3) finds in the data [56]; (ii) effect-size and uncertainty estimates derived from the top-performing model across all 40 models in the full model set; and (iii) model-averaged estimates of DP performance parameters (i.e., the clinical sensitivity and specificity of each DP in each tissue sample when samples are drawn from dogs in different clinical condition) and of *Leishmania* infection frequency, all with unconditional SEs [56].

## Results

### General descriptive and exploratory results

**Fig 2:**
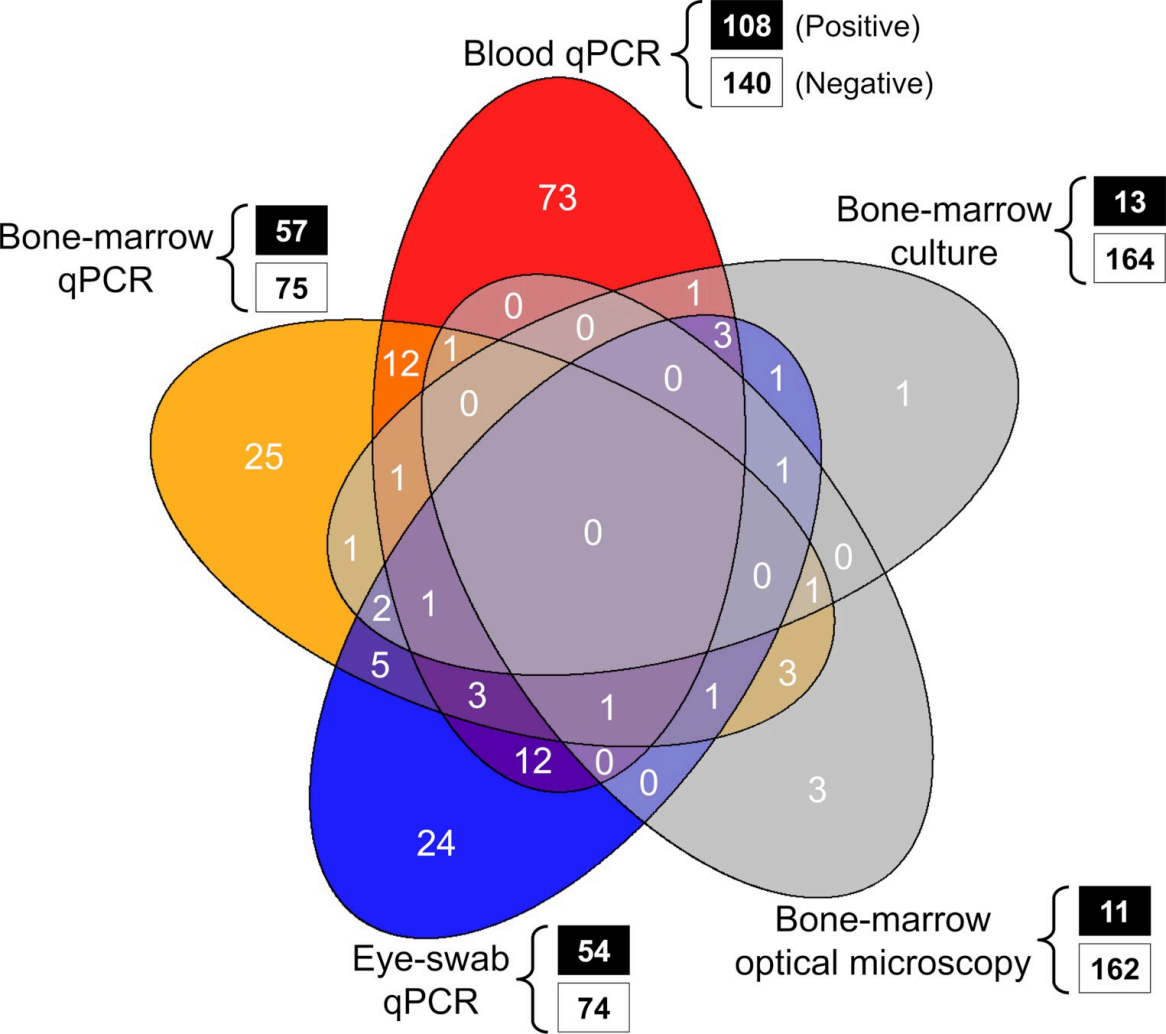
Patterns of positivity and co-positivity across five diagnostic procedures (parasitological and molecular) used for the detection of *Leishmania* infection in 294 domestic dogs.

We studied 294 dogs; the results of 1,366 individual diagnostic procedures (1,016 qPCR assays, 173 optical-microscopy examinations, and 177 bone-marrow cultures) were available for analysis (Fig 1 and Table 4). The full raw data are available in S1 Dataset. Overall, 176 of the dogs (59.86%) were positive in at least one procedure (Table 5). Molecular DPs yielded positive results more often than parasitological DPs both at the sample level (32.6 *vs*. 6.9%; Table 3) and at the dog level (60.1 *vs*. 12.4%; Table 5). Detailed results of duplicate qPCR assays show that in about 20% of dogs only one of the duplicate assays was scored as positive–and that, consequently, single-assay percent positivity (range, 29.7 to 34.9%) was lower (by about 10%) than that computed using data from both duplicate assays (42.2% for eye-swab, 43.2% for bone-marrow, and 43.6% for blood samples) (Table 5). Fig 2 shows the patterns of co-positivity across DPs; none of the 47 dogs tested by all five procedures was positive in all of them.

**Table 4:** Detecting *Leishmania* in dogs from central-western Brazil: Summary of test results stratified by diagnostic procedure and biological sample.

Diagnostic procedure	Biological sample	Tests run	Positive	%
Quantitative real-time PCR	Peripheral blood	496	160	32.26
	Bone-marrow aspirate	264	90	34.09
	Eye swab	256	81	31.64
(Subtotal molecular)		1,016	331	32.58
Optical microscopy	Bone-marrow aspirate	173	11	6.36
Parasite culture	Bone-marrow aspirate	177	13	7.34
(Subtotal parasitological)		350	24	6.86
Total		1,366	355	25.99

**Table 5:** Patterns of *Leishmania* detection/non-detection in dogs from central-western Brazil: Detailed results stratified by biological sample and diagnostic procedure.

Sample and procedure	Dogs tested	Outcome	Number	%
Blood qPCR	248	Any assay positive	108	43.55
		Both assays negative	140	56.45
		Both assays positive	52	20.97
		Only one assay positive	56	22.58
		First assay positive	81	32.66
		Second assay positive	79	31.85
Bone-marrow qPCR	132	Any assay positive	57	43.18
		Both assays negative	75	56.82
		Both assays positive	33	25.00
		Only one assay positive	24	18.18
		First assay positive	46	34.85
		Second assay positive	44	33.33
Eye-swab qPCR	128	Any assay positive	54	42.19
		Both assays negative	74	57.81
		Both assays positive	27	21.09
		Only one assay positive	27	21.09
		First assay positive	38	29.69
		Second assay positive	43	33.59
(Subtotal molecular)	286	(Any assay positive)	172	60.14
Bone-marrow microscopy	173	Test positive	11	6.36
		Test negative	162	93.64
Bone-marrow culture	177	Test positive	13	7.34
		Test negative	164	92.66
(Subtotal parasitological)	177	(Any test positive)	22	12.43
Total	294		176	59.86

qPCR, quantitative real-time PCR

**Fig 3:**
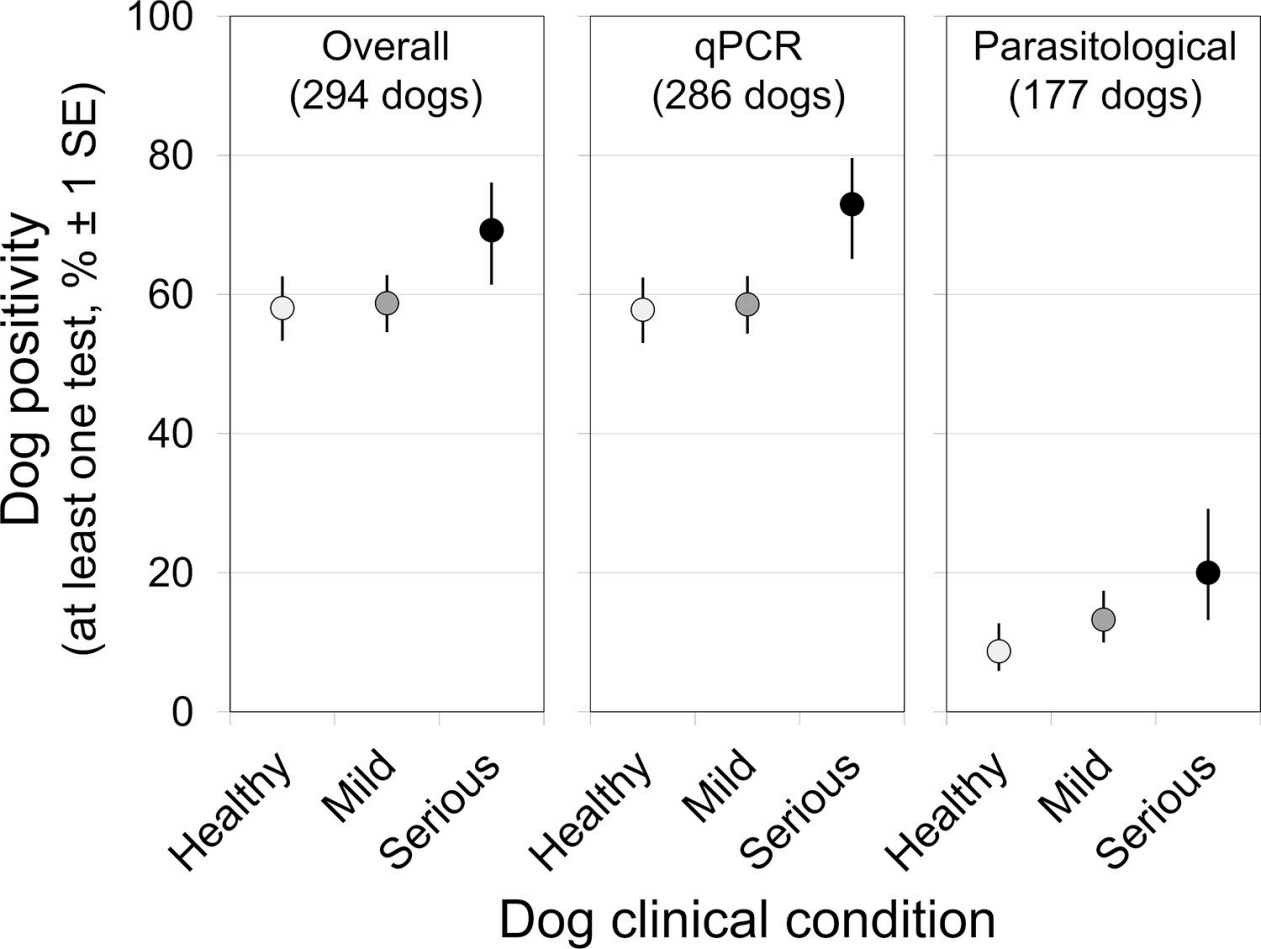
Positivity of diagnostic procedures used for the detection of *Leishmania* infection in domestic dogs of central-western Brazil: Predictions of exploratory bivariate models. Generalized linear models (binomial family, logit link function) were fitted to each of three databases (overall results, molecular procedures, and parasitological procedures) with clinical status as a three-level factor (healthy-looking, “Healthy”; mild disease, “Mild”; or serious disease, “Serious”); the number of dogs in each analysis is given in parentheses. Circles are stratum-specific marginal means predicted by each model, and error bars are standard errors (± 1 SE).

Table 6 summarizes the results of different DPs across the three strata of dog clinical condition. Overall, observed dog positivity (i.e., at least one DP positive) did not appear to vary much across clinical strata; although the odds of positivity appeared to be somewhat higher in seriously diseased dogs, the differences predicted by bivariate GLMs (binomial family, logit link) were within the range of estimation uncertainty (Fig 3). Within each clinical stratum, however, positivity was consistently much lower for parasitological than for molecular DPs (Table 6 and Fig 3).

**Table 6:** *Leishmania* detection/non-detection in dogs from central-western Brazil: Results stratified by biological sample/diagnostic procedure and dog clinical condition.

Sample/procedure	Dog clinical condition								
	Healthy-looking			Mildly diseased			Seriously diseased		
	Tested	Positive	%	Tested	Positive	%	Tested	Positive	%
Blood qPCR	89	37	41.57	125	53	42.40	34	18	52.94
Bone-marrow qPCR	52	22	42.31	64	30	46.88	16	5	31.25
Eye-swab qPCR	40	16	40.00	65	28	43.08	23	10	43.48
(Subtotal molecular)	109	63	57.80	140	82	58.57	37	27	72.97
Bone-marrow microscopy	69	4	5.80	81	6	7.41	23	1	4.35
Bone-marrow culture	69	2	2.90	83	6	7.23	25	5	20.00
(Subtotal parasitological)	69	6	8.70	83	11	13.25	25	5	20.00
Total	112	65	58.04	143	84	58.74	39	27	69.23

### Modeling

#### First modeling round

We first fitted the 11 multiple detection-state site-occupancy models presented in Table 2. The top-ranking model within this set was model {2} (see S1 and S2 Tables). This model represents the hypothesis that sensitivity differs between parasitological and molecular DPs, with all qPCRs having similar sensitivity and specificity; recall that 100% specificity is assumed for parasitological DPs (see Table 2). Model {2} estimates clinical sensitivity at 49.34% (CI 43.43–55.28%) for molecular DPs and at 11.30% (CI 7.44–16.36%) for parasitological DPs. In addition, model {2} estimates qPCR clinical specificity at 93.12% (CI 86.64–96.58%), suggesting that nearly 7% of positive qPCR assays were false positives. Model-averaged estimates and unconditional SEs computed across all models in this first set largely confirmed these results (S2 Table). The apparently low estimates of clinical sensitivity derived from these analyses were not unexpected, because *Leishmania* parasites or DNA are likely to be unavailable for detection in some samples drawn from infected dogs. As stressed above, these estimates refer to each whole diagnostic procedure–from the drawing of the sample to the scoring of the test. Specificity estimates consistently below 94% were however unexpected for our molecular DPs; as noted above, contamination with *Leishmania* DNA was the most likely explanation. To test this possibility, we built a qPCR plate-level “suspected contamination” covariate (“SC”, see **Methods**) and analyzed its effects on qPCR assay specificity. Table 7 summarizes qPCR results stratified by SC; it shows that the frequency of positive qPCR results was consistently higher for assays run on plates with suspected contamination (overall, 38.7%) than on plates without it (21.7%). A bivariate GLM (binomial family, logit link) suggests that the odds of assay positivity were, on average, 2.28 (CI, 1.70–3.05) times higher for qPCRs run on plates with suspected contamination. We therefore modeled SC effects in a second round of analyses (see S1 Table).

**Table 7:** Positivity of quantitative PCR (qPCR) assays run on samples drawn from central-western Brazilian dogs: Results stratified by biological sample and suspected qPCR plate contamination.

Sample	Suspected contamination in qPCR plate					
	No			Yes		
	Assays run	Positive	%	Assays run	Positive	%
Blood	214	44	20.56	282	116	41.13
Bone marrow	44	10	22.73	220	80	36.36
Eye swab	110	26	23.64	146	55	37.67
Total	368	80	21.74^a^	648	251	38.73^a^

^a^ The 95% confidence intervals (CIs) estimated by a generalized linear model were 17.81–26.25% for assays run on plates without evidence of contamination and 35.05–46.23% for assays run on plates with suspected contamination; note that the two CIs do not overlap

#### Second modeling round

Models including SC effects (see Table 2) clearly outperformed those fitted in our first round of analyses; the second-round top-ranking model (model {13}) had an AICc score 4.21 units smaller than the first-round top-ranking model (S1 Table). This is strongly suggestive of an important effect of qPCR plate-level contamination on the specificity of our molecular assays. Model {13} estimates a clearly positive effect of SC on the odds of getting a false-positive result (β_SC_ = 1.619, SE 0.634); this translates into a prediction of lower specificity for qPCR assays run on SC-positive plates (~82.5%) than for assays run on ‘clean’ plates (~96.0%; see Tables 8 and S2). Again, these results were overall consistent with model-averaged estimates and unconditional SEs computed across all models in the second-round set (S2 Table). We therefore used model {13} as the basis of a third round of analyses.

**Table 8:** Effects of suspected plate contamination on the specificity of quantitative PCR (qPCR) assays for the detection of *Leishmania* in dogs: Estimates from the top-ranking model in the second round of analyses (model {13}).

Parameter	Term	Estimate (%)	CI lower	CI upper
Sensitivity	Quantitative PCR	51.34	44.93	57.72
	Parasitological procedure	13.16	8.19	20.48
Specificity	Quantitative PCR–no contamination	95.96	87.58	98.77
	Quantitative PCR–suspected contamination	82.48	68.44	91.09
	Parasitological procedure	100 (fixed)	-	-
Infection	Mean infection frequency (intercept)	51.44	36.79	65.85

CI, 95% confidence interval (lower and upper limits)

#### Third modeling round

The two top-ranking models in the third round of analyses suggest that the sensitivity of our DPs varied depending on whether each dog was/was not seriously diseased (S1 Table). In particular, the top-performing model in this set (model {32}) suggests that the sensitivities of qPCR and OM may be somewhat lower in seriously diseased than in apparently healthy or mildly diseased dogs, whereas the opposite pattern emerges for BMC (Tables 9 and S2). However, effect-size estimates for qPCR (β_Serious disease-qPCR_ = −0.473, SE 0.289) and especially OM (β_Serious disease-OM_ = −0.375, SE 1.101) were fairly uncertain and effectively indistinguishable from zero; the positive BMC effect estimate was also uncertain but clearly larger (β_Serious disease-BMC_ = 1.657, SE 0.755). Model-averaged estimates from this third-round model set suggest that qPCR sensitivities were somewhat lower in seriously-diseased dogs, that OM sensitivities were very similar across clinical conditions, and that BMC sensitivity was higher (albeit with substantial uncertainty) in seriously-diseased dogs (see S2 Table). We therefore fitted a series of four additional models, based on model {32}, in which the “seriously diseased” covariate affected, respectively, BMC sensitivity only, parasitological DPs only, OM only, or qPCR only (Table 3).

**Table 9:** Effects of dog clinical status on the sensitivity of *Leishmania* diagnostic procedures: Estimates from the top-ranking model in the third round of analyses (model {32}).

Parameter	Term	Estimate (%)	CI lower	CI upper
Sensitivity	Quantitative PCR–no or mild disease	54.44	47.10	61.59
	Quantitative PCR–serious disease	42.68	31.06	55.16
	Bone-marrow microscopy–no or mild disease	12.28	7.22	20.11
	Bone-marrow microscopy–serious disease	8.78	1.12	44.90
	Bone-marrow culture–no or mild disease	12.28	7.22	20.11
	Bone-marrow culture–serious disease	42.34	14.12	76.63
Specificity	Quantitative PCR–no contamination	95.40	87.48	98.40
	Quantitative PCR–suspected contamination	80.51	67.94	88.96
	Parasitological procedure	100 (fixed)	-	-
Infection	Mean infection frequency (intercept)	47.87	33.42	62.68

CI, 95% confidence interval (lower and upper limits)

#### Final modeling round

The top-ranking model in the final model set was model {36} (S1 Table). We therefore found support for the hypothesis stating that clinical sensitivity differs between qPCRs and parasitological DPs; all qPCRs have similar sensitivities and specificities; qPCR specificity decreases by the same average amount in samples run on plates with suspected contamination; and the sensitivity of BMC (but not of other DPs) is higher in seriously diseased dogs (Tables 3 and S1). The second-ranking model also has support from the data (AICc 1.38 units larger than that of model {36}), and is the third-round top-ranking model summarized above (Tables 9 and S1). Table 10 shows the estimates of DP performance and *Leishmania* infection frequency derived from the top-performing model in our full model set. Note that the naïve infection frequency in our data (59.9%; Table 4) is about 1.22 times higher than the model-based estimate (49.1%; Table 10). The naïve calculation is based on the assumption that a dog was infected if at least one DP was positive, whereas the model-based estimate formally takes into account the imperfections of the detection process and the effects that both biological (dog clinical condition) and technical factors (suspected qPCR plate contamination) can have on that process (Table 10).

**Table 10:** Performance of diagnostic procedures for detecting *Leishmania* infection in dogs: Estimates from the top-ranking model (model {36}).

Parameter	Term	Estimate (%)	CI lower	CI upper
Sensitivity	Quantitative PCR	51.92	45.39	58.38
	Bone-marrow microscopy	11.68	6.91	19.07
	Bone-marrow culture–no or mild disease	11.68	6.91	19.07
	Bone-marrow culture–serious disease	44.47	14.65	78.89
Specificity	Quantitative PCR–no contamination	95.60	87.51	98.54
	Quantitative PCR–suspected contamination	80.93	67.55	89.64
	Parasitological procedure	100 (fixed)	-	-
Infection	Mean infection frequency (intercept)	49.10	34.24	64.12

CI, 95% confidence interval (lower and upper limits)

More complex versions of model {36} all had larger AICc scores than their simpler counterpart (see S1 Table). The first alternative model (AICc 1.89 units larger than {36}) estimated a near-zero coefficient for the effect of mild disease on BMC sensitivity (β_Mild disease-BMC_ = 0.249, SE 0.534); this supports the suggestion that clinical-condition effects were mainly driven by differences between seriously-diseased and other dogs, whether apparently healthy or mildly diseased. Similarly, versions of model {36} testing for clinical-condition effects on the odds of dog infection did not perform any better than model {36} itself; AICc scores were from 1.93 to 4.05 units larger (S1 Table). Two-level covariate models estimated near-zero effects for apparently healthy (−0.144, SE 0.353) and seriously diseased dogs (0.049, SE 0.598). A final alternative version of model {36} with a three-level clinical-condition covariate on infection also estimated near-zero effects for mildly-diseased (0.145, SE 0.362) and seriously-diseased dogs (0.141, SE 0.636) relative to apparently healthy dogs (see S1 Table). Overall clinical condition was, therefore, a poor predictor of *Leishmania*-infection status in our sample of dogs.

**Fig 4:**
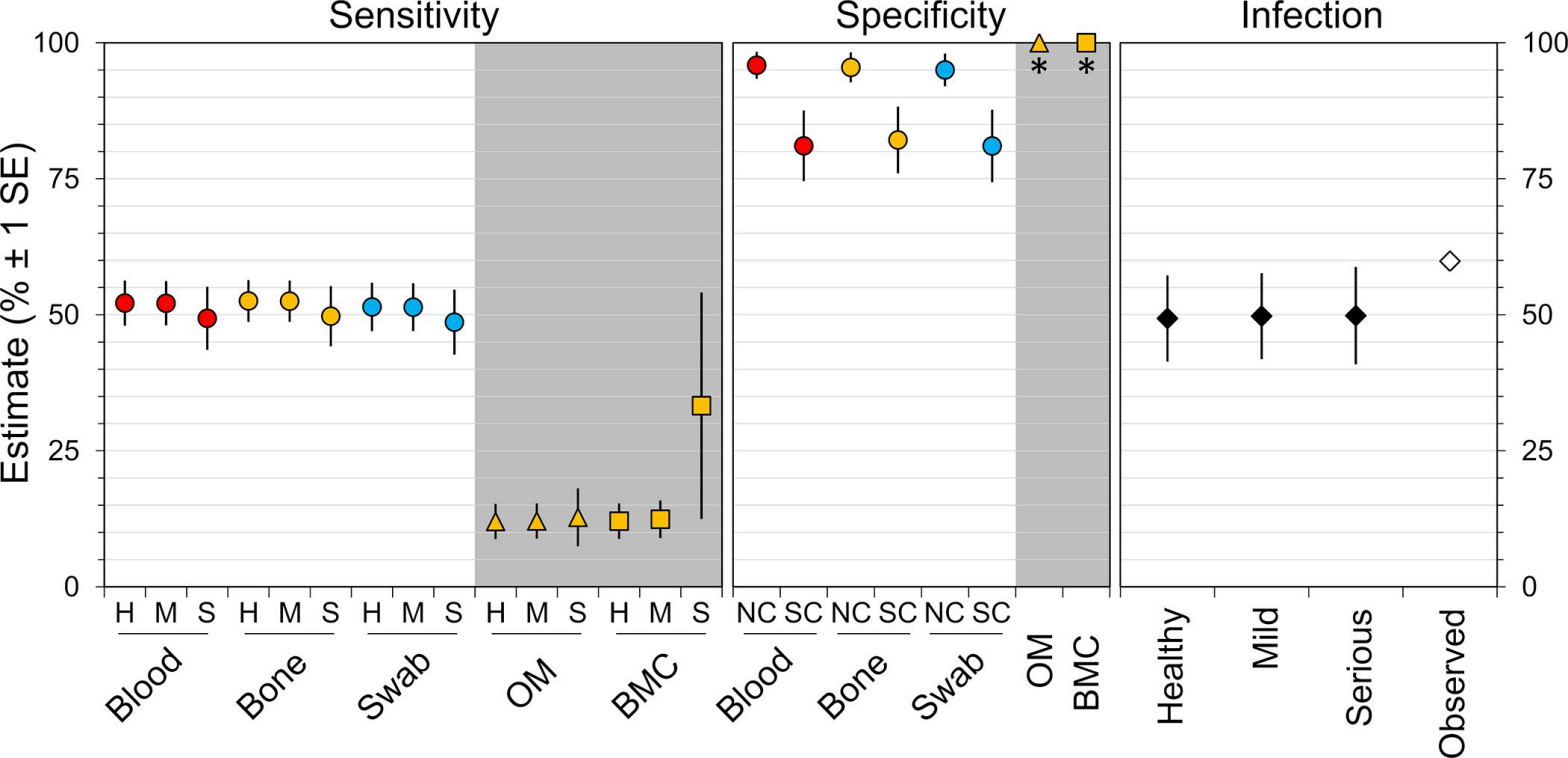
Detecting *Leishmania* in dogs: Performance of molecular and parasitological diagnostic procedures and corrected infection frequency. Point estimates are model-averaged values (expressed as percentages) derived from the full, 40-model set; error bars are unconditional standard errors (± 1 SE). All values correspond to a single procedure–one qPCR assay or one parasitological evaluation (in our case, two microscope-slide readings for optical microscopy and inverted optical microscope examination every two days over a 30-day period for parasite cultures). Circles represent molecular diagnostic procedures (quantitative PCR assays) run on different biological samples: peripheral blood (“Blood”, red), bone-marrow aspirates (“Bone”, orange), and eye swabs (“Swab”, blue). Parasitological diagnostic procedures (grey background) used bone-marrow aspirate samples (hence the orange color) and included optical microscopy (“OM”, triangles) and bone-marrow culture (“BMC”, squares); note that specificity values were fixed at 100% for OM and BMC (see asterisks). Diamonds represent infection-frequency estimates (black) and observed infection frequency (white; calculated as the percent of dogs with at least one positive result in any diagnostic procedure). Dogs in our sample were apparently healthy (“H” or “Healthy”), mildly diseased (“M” or “Mild”), or seriously diseased (“S” or “Serious”). The specificity of molecular diagnostic procedures was estimated for qPCR assays run on plates with no evidence of contamination (“NC”) or on plates with suspected contamination (“SC”).

Fig 4 presents model-averaged estimates of DP clinical sensitivity and specificity, along with *Leishmania* infection-frequency estimates, derived from the full, 40-model set; error bars are unconditional SEs [56] (see also S2 Table). Molecular-DP sensitivities were all estimated at ~50% (Fig 4). Parasitological-DP sensitivity estimates were much lower (~12%) for healthy-looking and mildly-diseased dogs, but rose to ~33.3% for BMC in seriously-diseased dogs; however, this latter point estimate was associated with substantial uncertainty (Fig 4). Specificity estimates were consistently above 95% for qPCR assays run on plates without signs of contamination, but dropped to ~81–82% when assays were run on plates with suspected contamination (Fig 4). Overall, we found little evidence of variation in qPCR performance across different biological samples; invasive (blood, bone marrow) and non-invasive sampling (eye swabs) led to comparable qPCR clinical sensitivities and specificities (Fig 4). Finally, model-averaged point estimates of *Leishmania* infection frequency varied very little (from 49.33 to 49.84%) across dog clinical-condition classes (Fig 4). These estimates were lower than the observed infection frequency of 59.86%, suggesting that about 30–31 of the 176 dogs classified as infected with the naïve criterion (at least one DP positive) were most likely uninfected.

## Discussion

Detecting pathogens in their hosts is a complex, hierarchical problem with major implications for individual and population health [11,12,19,20,43,57,58]. Particularly in field settings, the absence of fail-safe diagnostic procedures leads to uncertainty about how different pathogen-detection tests perform on different types of samples–and, consequently, to uncertainty about host-infection frequencies and their variation [19,20,31,43,57–61]. Explicitly accounting for the uncertainties inherent to pathogen detection could thus yield sounder, more realistic insight on diagnostic-test performance and infectious-disease epidemiology [11,12,43,57–59]. In this study, we illustrate how a hierarchical-modeling framework can help dissect the process of detecting a major human pathogen, *Leishmania infantum*, in its primary reservoir host, the domestic dog. Our estimates of clinical sensitivity were low for a widely-used kDNA qPCR-based procedure, and even lower for two parasitological tests; sensitivity, on the other hand, was similar for invasive and non-invasive biological samples and across dog clinical conditions. Importantly, qPCR specificity was overall acceptable (> 95%) only when very stringent criteria were used to identify signs of possible qPCR-plate contamination. After formally accounting for these multiple sources of uncertainty, we estimated dog-infection frequency at about 50%, again with little variation among dogs in different clinical conditions; infection with *Leishmania* was thus as widespread among apparently healthy dogs as it was among mildly- and seriously-diseased dogs in our sample.

Our analyses suggest that the sensitivity of qPCR targeting *Leishmania* kDNA was only about 52% (Table 10). This is apparently much lower than previous estimates; for example, Jara et al. [54] reported that a *Leishmania* kDNA-based qPCR was 97.9% sensitive when applied to human clinical samples. What underlies this difference? Importantly, Jara et al. [54] computed sensitivity as the percent of qPCR-positive samples among those in which a composite reference standard had yielded at least one positive result (see Table 1). If reference-standard tests were specific enough, this means that the samples used to compute the sensitivity of the index qPCR did all, or nearly all, contain the target for detection–*Leishmania* DNA [54] (see also, e.g., [62] and Table 1). In our study (as in most real-life settings), host-level infection and sample-level target availability were both uncertain. In these cases, a negative test result can stem from two distinct processes [11,12]. First, the target for detection may not be present in the sample. This can happen even if the sample comes from an infected host, reflecting the common fact that pathogens are not evenly distributed within their hosts–for example, pathogen tissue tropism induces spatial variation, and bursts of pathogen reproduction induce temporal variation. A negative test result can hardly be seen as a false negative when the sample did not contain the test’s target for detection–whatever the host’s infection status, the test gave the correct answer at the sample level [11,12,57,63,64]. Second, the test can fail to detect its target even when that target is present in the sample; this is a true false-negative result, and reflects the common fact that no test has perfect sensitivity [11,12,57,63,64]. In our study, there was no guarantee that all samples drawn from infected dogs did contain *Leishmania* parasites or their DNA. Therefore, our sensitivity estimates are estimates of what we call “clinical sensitivity”–they refer not to the test itself, but to the whole diagnostic procedure, from sampling to test scoring (Fig 1). Jara et al.’s [54] estimate is probably closer to the “true” sensitivity of *Leishmania* kDNA-based qPCR assays; what their approach cannot reveal, however, is how many samples did contain the target (so that the host was infected) but were negative in both the reference standard and the index tests. This is a serious, yet most often ignored, drawback of diagnostic-test evaluations based on the implausible assumption that “reference standards” are just perfect (Table 1).

A more formal statement of the pathogen-detection problem can shed light into its three key components–host-level infection, sample-level target availability and test-level sensitivity–and their relations [11]. Let Pr(+) be the probability that a pathogen-detection test yields a positive result when run on a given sample drawn from a given host. Pr(+) depends on (i) the probability that the host is infected (denoted Ψ); (ii) the probability that the detection target is present in the sample, given host infection (denoted θ); and (iii) the probability that the target is detected by the test, given target presence in the sample and, hence, host infection (the true sensitivity of the test, denoted *p*) [11]. Specifically, Pr(+) = Ψ × θ × *p*. When the host is infected (Ψ = 1.0), the probability of getting a positive test result is Pr(+) = θ × *p*, and this is what the estimates of clinical sensitivity in Table 10 stand for. Using Jara et al.’s [54] estimate of kDNA qPCR “true” sensitivity (*p* ≈ 0.98) and our best estimate of kDNA qPCR clinical sensitivity (~0.52; Table 10) in this equation, the probability of getting a positive qPCR out of a sample drawn from an infected dog would be Pr(+) ≈ 1.0 × θ × 0.98 ≈ 0.52; therefore, θ ≈ 0.52 / 0.98 ≈ 0.53. This suggests that, if our kDNA qPCR had a “true” test sensitivity similar to that reported by Jara et al. [54], then *Leishmania* DNA was most likely absent from nearly half (~47%) of the samples we drew from *Leishmania*-infected dogs (and had amplifiable DNA; see Fig 1). For a mean qPCR assay sensitivity of ~95%, our clinical sensitivity estimate would suggest that only ~55% of those samples contained *Leishmania* DNA. Our estimates of parasitological-DP clinical sensitivities, on the other hand, suggest that, if θ was ~55%, then the “true” sensitivity of MO and BMC in apparently healthy or mildly diseased dogs was *p* ≈ 0.12 / 0.55 ≈ 0.22, or ~22%; in seriously-diseased dogs, BMC would have *p* ≈ 82%–with the caveat of a very large uncertainty (Table 10, Fig 4).

It is important to note that θ and *p* are also confounded whenever reference-standard tests or procedures are applied not to the actual samples used for assessing the index test(s), but to the hosts (Table 1). For example, Gomes et al. [65] used clinical, immunological, and parasitological reference-standard criteria to classify patients (not samples) as “with” or “without” American tegumentary leishmaniasis (ATL), and then computed qPCR sensitivity as the percent of samples drawn from patients “with” ATL that yielded positive qPCR results [65]. Their (clinical) sensitivity estimates ranged from 54.7% to 75.0%, depending on sample type (lesion biopsy or lesion swab) and qPCR chemistry (TaqMan or SYBR Green) [65]–which, not surprisingly, is more in line with our present results. If we assume that the “true” mean sensitivity of their qPCR was *p* ≈ 95% (and specificity ~100%), then we can infer that *Leishmania* DNA was likely present in about 78–79% of biopsy and swab samples taken from skin lesions of ATL patients.

Studies with experimentally infected hosts provide further insight into the clinical sensitivity of diagnostic procedures (Table 1). Because host-level infection is all but certain (i.e., Ψ ≈ 1.0; but see Table 1), the proportion of positive tests effectively measures θ × *p*, just as in the present report. For example, Rodríguez-Cortés et al. [31] reported TaqMan kDNA qPCR clinical sensitivities of 50% for blood samples and 58% for bone-marrow samples, and Abbehusen et al. [66] found 42% clinical sensitivity for both spleen-aspirate and skin-biopsy samples, but 100% for lymph-node aspirates [66] (see also [67]). Assuming again that “true” qPCR-assay sensitivity was *p* ≈ 95% (and specificity 100%) in all cases, these results would suggest that *Leishmania* DNA was likely available for detection, on average, in ~52% of blood samples, ~61% of bone-marrow samples, ~44% of spleen and skin samples, and ~100% of lymph-node aspirates. Under the same assumptions, longitudinal data from Cantos-Barreda et al. [29] would suggest that availability ranged from ~44 to 79% in saliva and from ~79 to 95% in bone marrow, depending on the time elapsed between infection and sampling.

In spite of the obvious interest of the matter, we could not estimate θ and *p* separately with our data. This was because the ‘standard’ three-level hierarchical models [63] implemented in Presence 2.13.11 [55] assume perfect test specificity–the dataset must contain no false-positive results [11,12,63] (but see below). While we could safely assume 100% specificity for parasitological tests (OM and BMC) run by expert staff, our initial analyses revealed that about 7% of all positive qPCR results were most likely false-positives. This led us to look for, and discover, subtle signs of possible contamination in some qPCR plates; we modeled this source of heterogeneity with a “suspected contamination” covariate, but the results still suggested that specificity was too low (~95 to 96%; Tables 8–10) to justify the 100% assumption even for qPCRs run in apparently contamination-free plates. Since samples were not available for retesting, we decided to focus on two-level models in which specificity can be estimated from the data but the two component parts of clinical sensitivity (θ and *p*) cannot be separated. Thus, while the low estimates of clinical sensitivity we present do suggest, together with published qPCR sensitivities (e.g., [31,54,62,65–67]), that many blood, bone-marrow, and eye-swab samples taken from *Leishmania*-infected dogs may not contain parasites or their DNA, formal estimation of θ will require further, focused work [11,12]. We note that multilevel (Ψ, θ, *p*) models allowing for false-positive errors are structurally non-identifiable [68]. Overcoming identifiability issues (an area of active research, particularly in the context of environmental DNA studies [64,68,69]) requires both (i) using at least one survey method that yields no false-positive results and (ii) running independent calibration experiments to generate auxiliary data [64,68]. A recently developed Bayesian implementation [69] requires, in turn, (i) specifying informative prior distributions for all model parameters and (ii) imposing restrictions on some parameter values–such as, e.g., that, for both θ and *p*, the probability of a true positive be larger than the probability of a false positive [69]. Our clinical-sensitivity estimates, in any case, are valuable in that they reflect what veterinarians or researchers would see if they ran one diagnostic test on a sample drawn from a dog of unknown infection status. Whatever the relative contributions of θ and *p*, our results suggest that a strategy based on a single parasitological test will miss about 88% of infections in apparently healthy or mildly-diseased dogs; bone-marrow culture is likely to perform better in seriously-diseased dogs, but over half of infections will still be missed (Table 10). Similarly, a strategy based on a single kDNA qPCR assay will likely miss nearly half of infections–either because of lack of *Leishmania* DNA in the sample or, less often, because of the test failing to amplify its target.

We found similar overall performance of kDNA qPCR for invasive (blood, bone-marrow) and non-invasive (eye-swab) samples (Tables 3 and 10, Fig 4). Because eye swabbing is cheaper, easier, and safer than blood or bone-marrow sampling, this seems to add support to the view that eye swabbing may be useful in *Leishmania*-infection surveys (e.g., [4,70–72]). We note, however, that to compute our qPCR performance estimates we excluded all the samples in which a PCR targeting the dogs’ *β-actin* gene did not yield any band, which indicates that those samples did not contain amplifiable DNA after extraction (Fig 1). As Fig 1 shows, this was more frequent in eye-swab samples (53/268; 19.8%) than in either blood (7/294; 2.4%) of bone-marrow samples (9/173; 5.2%). Devising strategies to increase the odds of getting enough good-quality DNA from eye swabbing–by improving sampling protocols, DNA-extraction procedures, or both–thus emerges as a key topic for future research. Our results indicate that, when good-quality DNA is available in the sample, kDNA qPCR should perform similarly (with a clinical sensitivity of ~52% and a specificity of ~96% per assay) in blood, bone-marrow, and eye-swab samples. Other samples, whether invasive (e.g., spleen or lymph-node aspirates) or non-invasive (e.g., oral, nasal, or vaginal swabs), may yield different clinical sensitivities depending, for a given diagnostic PCR, on the sample-specific availability of *Leishmania* DNA.

After accounting for these many sources of diagnostic uncertainty, we derived a maximum-likelihood estimate of dog-infection frequency (~49.1%, or ~144–145 of the dogs in our sample; Table 10) that is moderately but clearly lower than the observed frequency (59.9%, or 176 dogs; Table 5). This suggests that, due to the vagaries of the pathogen-detection process, about 31–32 of our 294 study dogs would have been misclassified as infected with *Leishmania* by the oft-used tactic of declaring infection when at least one test result is positive. This upward bias may not only distort our understanding of *Leishmania* epidemiology and epizootiology; in addition, false-positive healthy dogs may be needlessly treated with anti-*Leishmania* drugs–or even culled. On the other hand, Table 5 shows that any single-test strategy would have grossly underestimated the true frequency of infection. For example, of the 248 dogs tested by blood qPCR, just 81 (32.7%) were positive in the first assay; assuming that the true frequency of infection in this dog subset was as estimated by our top-ranking model (49.1%), this means that about 41 *Leishmania*-infected dogs would have been missed with a one-blood-qPCR strategy. Similarly, a strategy based on a single eye-swab qPCR would have missed between 20 (first assay) and 25 (second assay) infections among the 128 dogs tested by this procedure (Table 5). Single parasitological tests would miss over 85% of infections (Table 5 and S1 Dataset).

## Conclusions and outlook

This study illustrates how hierarchical site-occupancy models can sharpen our understanding of the complex problem of diagnosing host infections with hard-to-detect pathogens like *Leishmania* spp. First, hierarchical modeling was instrumental to discovering (and formally accounting for) subtle signs of possible qPCR-assay contamination. Our results suggest that negative/blank controls should only be scored as negative when amplification signals appear at Ct ≥ 40 cycles and the melting curves do not match those expected for *Leishmania*; otherwise, samples should be retested. Second, our approach allowed us to derive statistical estimates of key performance parameters for five diagnostic procedures used to detect *Leishmania* parasites or their DNA in dogs and other hosts. Low clinical sensitivies likely reflect the absence of parasites/DNA in perhaps ~50–70% of samples drawn from infected dogs; separate, formal estimation of sample-level target availability and test-level “true” sensitivity emerges as a major topic for future research [11,12,68,69]. We also found evidence suggesting that qPCR can perform similarly on invasive (blood, bone marrow) and non-invasive (eye-swab) samples; the latter, however, were overall less likely to contain amplifiable DNA, and fully exploiting the practical advantages of non-invasive eye-swabbing will require better DNA sampling/extraction protocols. Finally, our approach yields estimates of host-infection frequency that formally account for the imperfections of the diagnostic process. The results show how assuming perfect test performance can lead to biased conclusions–ranging from gross underestimation to moderate overestimation of infection frequencies, depending on what testing-scoring strategy is chosen to declare infection. Naïve approaches based on implausible assumptions about diagnostic performance are unlikely to help us understand the patterns, drivers, and dynamics of *Leishmania* infection in dogs. More generally, this report adds to the growing body of evidence (e.g., [11,12,19,20,41–43,57]) suggesting that site-occupancy modeling can contribute crucial tools for the study of infectious-disease ecology and epidemiology.

## Supporting information

S1 Protocol DNA extraction from eye-swab samples. (PDF)

S1 Dataset Raw data. The dataset includes test results (0 = negative with ambiguity; 1 = positive with ambiguity; 2 = positive without ambiguity; dash = test not run) and covariate values (0 = no; 1 = yes).
(XLSX)

S1 Table Model sets considered in each modeling round. For each model, the table presents model structure (for infection, Ψ; sensitivity, p_11_; and false-positive rate, p_10_); AICc scores (second-order Akaike information criterion); ΔAICc (variation in AICc relative to the top-ranking model); AICw (Akaike weights); number of estimable parameters (k); and deviance (−2 × log-likelihood).
(XLSX)

S2 Table Parameter estimates from each modeling round: top-model estimates and model-averaged estimates. Estimates are given on the probability scale, together with standard errors (SEs); note that SEs for model-averaged estimates are unconditional SEs.
(XLSX)
