# Supplementary material for: Detecting Leishmania in dogs: A hierarchical-modeling approach to investigate the performance of parasitological and qPCR-based diagnostic procedures
Source: PLoS Negl Trop Dis. 2022 Dec 16;16(12):e0011011. doi: 10.1371/journal.pntd.0011011 (PMC9803295; doi:10.1371/journal.pntd.0011011)
Supplement: S1 Protocol — (PDF) [file pntd.0011011.s001.pdf]

S1 Protocol. DNA extraction from eye-swab samples

Eye-swab samples samples were maintained at −20ºC until extraction with Biopur® Mini Spin Plus kits (Biometrix, Atuba, Brazil), with minor modifications to the manufacturer's protocol, as follows:

Sample lysis

- Add 200 µL of Lysis Buffer plus 20 µL of Proteinase K (20 mg/mL) to the 1.5 mL microtube containing each sample; vortex the tube vigorously (3 times) and incubate the solution at 56ºC for 30 minutes.
- Remove the fluid (lysate) from the cotton tip by strongly pressing the tip against the inner tube wall; centrifuge at 13,000 g for 1 minute at room temperature.

DNA binding

- Add 400 µL of Ligation Buffer to the lysate, homogenize the mixture by vortexing and transfer lysate to the kit DNA-adsorption column, where DNA is adsorbed onto a silica-gel membrane.
- Place the column in a sterile Eppendorf tube, leave standing for 1 minute at room temperature, and centrifuge at 13,000 g for 1 minute at room temperature; discard flow-through.

Membrane washing-drying

- Add 500 µL of Wash Buffer I; centrifuge at 13,000 g for 1 minute at room temperature; discard flow-through.
- Add 800 µL of Wash Buffer II; centrifuge at 13,000 g for 1 minute at room temperature; discard flow-through.
- Dry the silica membrane by centrifugation at 13,000 g for an extra 4 minutes at room temperature without reagents.

DNA elution

- Transfer the column to a new, sterile Epperndorf tube and add 50 µL of 56 ºC-preheated Elution Buffer; incubate at room temperature for 5 minutes and centrifuge at 13,000 g for 4 minutes at room temperature; discard column.
- Store the DNA-cotaining flow-through at −20 ºC until use.
